# Adolescent PCOS and long-term metabolic risk: insights from triglycerides to high-density lipoprotein cholesterol ratio and high-density lipoprotein cholesterol profiles

**DOI:** 10.3389/fendo.2025.1579217

**Published:** 2025-06-03

**Authors:** Maria Nowak-Ciołek, Julia Jagoda Stachowiak, Katarzyna Krok, Julia Sokal, Żaneta Malczyk, Karolina Skrzyńska, Agnieszka Zachurzok

**Affiliations:** ^1^ Students’ Scientific Association at the Department of Pediatrics, Faculty of Medical Sciences in Zabrze, Medical University of Silesia in Katowice, Zabrze, Poland; ^2^ Department of Pediatrics, Faculty of Medical Sciences in Zabrze, Medical University of Silesia in Katowice, Zabrze, Poland; ^3^ Department of Pediatrics and Pediatric Endocrinology, Faculty of Medical Sciences in Katowice, Medical University of Silesia, Katowice, Poland

**Keywords:** pcos, adolescence, metabolic health, dyslipidemia, insulin resistance

## Abstract

**Introduction:**

Polycystic ovary syndrome (PCOS) is a complex endocrine disorder affecting 6–22% of women worldwide, with adolescent diagnosis posing significant challenges due to physiological differences between adolescents and adults. Historically, the application of the Rotterdam criteria in adolescent girls led to overdiagnosis, prompting the introduction of revised adolescent-specific criteria in 2017. These updated criteria require the presence of both irregular menstrual cycles and hyperandrogenism while excluding criterion of polycystic ovarian morphology. This study aims to assess whether a PCOS diagnosis based on adolescent-specific criteria correlates with an increased risk of metabolic disturbances in adulthood.

**Methods:**

A retrospective analysis was conducted on medical records of 34 adolescent patients diagnosed with PCOS according to the Rotterdam criteria, who were divided into a study group (SG, n=23) meeting the updated adolescent PCOS criteria and a comparison group (CG, n=11) that did not meet these criteria. After the mean follow-up of 5.5 years, they were reassessed based on clinical and metabolic parameters. Clinical assessments included body mass index (BMI), Ferriman-Gallwey scale, and body composition analysis. Biochemical analyses involved fasting lipid profiles, glucose metabolism markers, and insulin resistance indices.

**Results:**

Application of the updated diagnostic criteria resulted in a 33% reduction in PCOS diagnoses among adolescents. No significant differences in BMI were observed between SG and CG on the first and second visits. The triglycerides (TG) to HDL-cholesterol (HDL-C) ratio was significantly higher in the SG across both time points (p=0.029 in adolescence, p=0.049 in adulthood), correlating positively with insulin levels during adolescence (p=0.005, R=0.61). Additionally, HDL-C levels were consistently lower in the SG than in CG (p=0.025 in adolescence, p=0.033 in adulthood).

**Conclusion:**

The findings of this study suggest that the revised PCOS criteria in adolescence may assist in identifying individuals at risk of long-term metabolic abnormalities. Elevated TG/HDL-C ratios and persistent HDL-C abnormalities observed in the study group may indicate a metabolic predisposition independent of obesity. These preliminary results emphasize the potential importance of early metabolic monitoring in adolescent PCOS patients; however, they should be interpreted with caution and confirmed in larger cohorts to ensure sufficient statistical power and broader generalizability.

## 1 Introduction

Polycystic ovary syndrome (PCOS) is a complex endocrine disorder affecting 6–22% of women globally (1). In adult women, the primary diagnostic criteria for PCOS are the Rotterdam criteria, which require the presence of at least two of the following three features: oligo- or anovulation, clinical and/or biochemical hyperandrogenism, and polycystic ovarian morphology on ultrasound. Based on these symptoms, four phenotypes – A, B, C and D - were described. Phenotype A includes hyperandrogenism, menstrual irregularities, and ovarian cysts; Phenotype B presents with hyperandrogenism and cycle disturbances; Phenotype C involves hyperandrogenism and ovarian cysts; and Phenotype D is characterized by disrupted menstruation and cystic ovaries (2). The Rotterdam criteria, established in 2003, used to be applied to diagnose PCOS in adolescent girls, which has contributed to overdiagnosis in this population (3). However, relevant physiological differences exist between adolescents and adult women. Menstrual irregularities that are abnormal in adults are often considered normal variations in adolescents. In adolescents, the menstrual cycle length can vary between 21–45 days for up to three years post-menarche, while in adult women, the normal cycle range is 21–35 days. Irregular menstrual bleeding and anovulatory cycles within two years of menarche are typical due to the immaturity of the hypothalamic-pituitary-ovarian axis (4). Multifollicular ovarian morphology is also common in adolescent girls. This ovarian appearance results from heightened gonadotropin stimulation, leading to increased ovarian volume and follicle count. As a result, ovarian ultrasound assessment for diagnosing PCOS in adolescent girls is challenging and is generally discouraged for up to eight years post-menarche (5). First in 2017, and then in 2023, updated diagnostic criteria for PCOS in adolescent girls were introduced. These criteria specify the need for both irregular menstrual cycles and hyperandrogenism, excluding polycystic ovarian morphology as a criterion (6, 7). According to these updated criteria, a diagnosis of PCOS in adolescent girls can only be made if they meet the requirements for Rotterdam phenotype B or A.

PCOS diagnosis is associated with an increased risk of obesity or overweight, glucose intolerance, diabetes, dyslipidemia, and metabolic-associated fatty liver disease (8). The revised diagnostic criteria for PCOS in adolescents are expected to reduce overdiagnosis while identifying individuals at the highest long-term risk for metabolic disorders.

This study aims to assess whether a PCOS diagnosis based on adolescent-specific criteria correlates with an increased risk of metabolic abnormalities in adulthood.

## 2 Materials and methods

A retrospective analysis of the medical records of adolescent patients diagnosed with PCOS according to Rotterdam criteria was conducted. We searched for data including information about: age, weight, height, body mass index (BMI), regularity of menstrual cycle, pelvis ultrasound examination results, hirsutism assessment in Ferriman-Gallwey Scale, blood tests results: testosterone, 17OH progesterone (17OHP), dehydroepiandrosterone sulfate (DHEAS), androstenedione, aspartate aminotransferase (AST), alanine aminotransferase (ALT), total cholesterol (TC), LDL-cholesterol (LDL-C), HDL-cholesterol (HDL-C), triglycerides (TG), glucose and insulin.

Exclusion criteria included: eating disorders, Cushing’s syndrome diagnosis, adrenal cortex dysfunction (excluded on the basis of basal 17OHP <2ng/dl or ACTH analogue stimulated 17OHP <10ng/dl), diagnosis of adrenal or ovarian neoplasms, uncontrolled thyroid dysfunction (hypothyroidism or hyperthyroidism), hyperprolactinemia (concentration of prolactin ≥ 36 µg/l) and pregnancy.

Subsequently, after 4–11 years (median: 5.5 years) from the first visit, 34 young adult women were reassessed and assigned to two groups:

- The study group (SG) consisted of 23 young women aged between 18 and 25 years (mean: 21.6 ± 2.4 years). As adolescents, out of 23 patients 6 met the criteria for Rotterdam’s phenotype A, while remaining 17 were classified as phenotype B. All of the patients fulfilled the new diagnostic criteria for adolescents outlined below:
  1. Gynecological age above 2 years
  2. Specific criteria for menstrual disorders (6)
  3. Hyperandrogenism
    - biochemical: elevated levels of testosterone and/or 17OHP, androstenedione, DHEAS
    - clinical: hirsutism (Ferriman-Gallwey Score ≥ 8 points)
- The comparison group (CG) consisted of 11 young women aged between 20 and 26 (mean: 22 ± 1.9 years). In this group, as adolescents 2 girls with Rotterdam’s phenotype C and 5 with phenotype D were identified. Remaining 4 adolescents were considered as “at risk of PCOS”, because of symptoms and exclusion of other illnesses.

All patients’ phenotypes are presented in **Table 1**.

**Table 1:** Symptoms and associated phenotypes present in patients during adolescence.

Group	Symptoms/ Rotterdam’s phenotype	HA	MD	PCOM	Number of adolescent patients
**SG (n=23)**	A	**+**	**+**	+	6/34
	B	**+**	**+**		17/34
**CG (n=11)**	C	+		+	2/34
	D		+	+	5/34
	Not classified		+		2/34
	Not classified	+			2/34
	Summary	27/34 (78%)	30/34 (88%)	13/34 (38%)	

Patients overlapping Rotterdam and new criteria are highlighted in blue. SG, study group; CG, comparison group; HA, hyperandrogenism; MD, menstrual disorders; PCOM, polycystic ovary morphology.

At the second examination, patients completed the questionnaire about length and regularity of the menstrual cycle. In all subjects, physical examination was performed including measurement of height and weight, self-assessment of hirsutism using the Ferriman-Gallwey scale, and assessment of body composition using the bioimpedance method (Tanita MC-780MA-N).

Lipids (HDL-C, LDL-C, TC, TG), carbohydrates parameters (glucose, insulin), liver enzymes (AST, ALT) were assessed in the fasting stat (all patients were informed about the requirement to fast for 8 to 12 hours prior to blood sample collection, and fasting status was confirmed on the morning of the blood draw).

We calculated parameters related to insulin resistance (IR):

- HOMA-IR (homeostatic model assessment for insulin resistance): [fasting glucose (mmol/l)] × [fasting insulin (mIU/l)]/22.5
- TyG index: ln[fasting triglycerides (mg/dl) × fasting glucose (mg/dl)/2]
- TyG-BMI: TyG index × BMI (kg/m^2^)
- TG/HDL-C: fasting triglycerides (mg/dl)/HDL-cholesterol (mg/dl)

Numerous cut-off values have been proposed for the indices evaluated in this study. However, with a focus on metabolic outcomes, we adopted the threshold of TG/HDL-C ≥ 3 as recommended by McLaughlin et al., who associated this value with the identification of insulin resistance and metabolic syndrome (9). For the TyG and TyG-BMI indices, we applied cut-off values established in prior research as indicative of elevated insulin resistance risk in females, specifically 7.94 for the TyG index and 168.64 for TyG-BMI (10). For HOMA-IR, we used the cut-off value 2.1 proposed by Biernacka-Bartnik et al., as it was derived specifically from a cohort of female patients with PCOS. Informed consent was obtained from all participants prior to their inclusion in the study, with each patient being fully informed of the study’s purpose, procedures, potential risks, and benefits, in accordance with ethical guidelines and institutional regulations. The study was conducted according to the Helsinki declaration and approved by the Local Ethics Committee (approval No BNW/NWN/0052/KB1/40/23).

Statistical analysis was conducted using Statistica 13.3 PL software. The SG and CG were compared using either the Mann-Whitney U-Test or the Student’s t-test for independent samples, depending on data distribution as assessed by the Shapiro-Wilk test. To examine the correlation, Spearman’s rank correlation test was used. The Fisher’s exact test was used to determine if there was a significant difference between proportions in groups. Group comparisons adjusted for covariates were performed using analysis of covariance (ANCOVA), with transformations applied where necessary to meet model assumptions. Due to the relatively small sample size of the patient group, *post hoc* power analyses were performed to assess the likelihood of detecting significant effects given the observed effect sizes and sample distributions. The Bonferroni correction was not applied in this study due to its exploratory nature, the small sample size, and the primary aim of identifying potential metabolic risk signals requiring confirmation in larger populations.

## 3 Results

At the first visit, in our cohort, 27 (78%) participants presented with hyperandrogenism (HA), 30 (88%) with menstrual disorders (MD), and 13 (38%) with polycystic ovarian morphology (PCOM). Applying the updated diagnostic criteria for adolescents resulted in a 33% reduction in PCOS diagnoses among the study participants so finally SG comprised 23 and CG – 11 participants.

Statistical analysis showed no significant differences in baseline characteristics, including age and BMI, between the groups. However, differences were observed in testosterone and DHEAS levels during adolescence (**Table 2**).

**Table 2:** Clinical and biochemical characteristics of patients in the study and comparison groups at the time of diagnosis and at the time of follow-up visit.

Clinical features	Study group	Comparison group	p value
ADOLESCENCE			
Age (years)	16.0 [15.0 – 17.0]	16.0 [16.0 – 17.0]	0.24**
BMI (kg/m^2^)	27.1±6.3	26.4±5.5	0.78*
Number of patients with obesity	7.0 (30.4%)	3.0 (27.3%)	0.38***
Number of patients with Ferriman-Gallwey Score ≥8	7.0 (30.4%)	2.0 (18.2%)	0.59***
Testosterone (ng/dl)	67.2±23.9	41.2±23.6	0.009*
DHEAS (μg/dl)	412.4±161.4	280.9±106.4	0.028*
ADULTHOOD			
Age (years)	21.6±2.4	22±1.9	0.60*
Median duration of illness (years)	5.0 [4.0 – 7.0]	6.0 [4.0 – 7.0]	1.0**
BMI (kg/m^2^)	26.3 [21.6 – 34.0]	26.9 [21.3 – 32.6]	0.83**
Number of patients with obesity	9.0 (39.1%)	3.0 (27.3%)	0.39***
Body mass (kg)	77.4±24.3	73.3±17.5	0.62*
Muscle mass (kg)	47.9±8.8	47.4±5.2	0.87*
Fat mass (kg)	19.3 [15.4 – 35.1]	18.3 [15.4 – 35.0]	0.65**
Free fat mass (kg)	50.5±9.2	50.0±5.5	0.87*

Group differences were analyzed using the Student’s t-test (*) for normally distributed variables (presented as mean ± standard deviation), the Mann–Whitney U test (**) for non-normally distributed variables (presented as median and interquartile range, 25th–75th percentile), or the Fisher’s exact test (***) for categorical data. DHEAS, dehydroepiandrosterone sulfate; BMI, body mass index.

Comparison of biochemical parameters (**Tables 3** and **4**) revealed a statistically significant difference in HDL-C levels, which were decreased in the study group compared to the comparison and in the TG/HDL-C index, which was higher in the SG. These findings were consistent across both adolescence and adulthood. In the ANCOVA model with log-transformed TG/HDL-C as the dependent variable, and BMI as a covariate, group affiliation showed a statistically significant effect in both adolescents (F(1.18) = 6.68; p = 0.019) and adults (F(1.29) = 4.76; p = 0.037). In adolescents, BMI did not significantly contribute to the model (p = 0.39), indicating that the group effect was independent of BMI, whereas in adults, BMI was additionally found to be a significant predictor (p = 0.044), while fat mass remained non-significant (p = 0.27).Interestingly, TG/HDL-C positively correlated with insulin levels but only during adolescence (p=0.005, R=0.61).

**Table 3:** Biochemical characteristics of patients in the study and comparison groups during adolescence.

Parameters	Study group	Comparison group	p value
AST (μ/l)	16.8 [14.6 – 20.6]	15.6 [14.6 – 16.3]	0.28**
ALT(μ/l)	11.95 [9.8 – 14.0]	11.55 [10.0 – 12.0]	0.77**
TC (mg/dl)	162±31.3	150.4±35.6	0.45*
LDL-C (mg/dl)	93.0 [77.0 – 104.0]	76.4 [65.8 – 78.0]	0.27*
HDL-C (mg/dl)	43.2±9.4	52.6±5.7	0.025* 0.037^#^
TG (mg/dl)	133.7 [86.0 – 164.0]	74.0 [73.0 – 104.0]	0.17**
Glucose (mg/dl)	81.0 [79.0 – 86.0]	90.5 [84.3 – 98.7]	0.064**
Insulin (mIU/ml)	13.0 [9.4 – 16.3]	11.4 [6.1 – 12.0]	0.20**
TG/HDL-C	2.9 [1.9 – 4.0]	1.48 [1.2 – 2.0]	0.029** 0.019^#^
TyG	8.5±0.5	8.3±0.5	0.55*
TyG-BMI	243.0±56.0	228.7±43.8	0.58*
HOMA-IR	2.8 [1.9 – 3.4]	2.7 [1.4 – 3.4]	0.62**

Group differences were analyzed using the Student’s t-test (*) for normally distributed variables (presented as mean ± standard deviation) and the Mann–Whitney U test (**) for non-normally distributed variables (presented as median and interquartile range, 25th–75th percentile). p-values (#) were adjusted for BMI and fat mass using analysis of covariance (ANCOVA), with log-transformation of the dependent variable applied to meet model assumptions. AST, aspartate aminotransferase; ALT, alanine aminotransferase; TC, total cholesterol; LDL-C, LDL-cholesterol; HDL-C, HDL-cholesterol; TG – triglycerides; TG/HDL-C – triglycerides to HDL-cholesterol index; TyG – triglycerides glucose index; TyG-BMI – triglycerides glucose-BMI index; HOMA-IR, homeostatic model assessment for insulin resistance.

**Table 4:** Biochemical characteristics of patients in the study and comparison groups during adulthood.

Parameters	Study group	Comparison group	p value
AST (μ/l)	22.4±5.9	19.3±3.3	0.066*
ALT(μ/l)	17.0 [12.0 – 27.0]	17.0 [14.0 – 21.0]	0.76**
TC (mg/dl)	183.6±32.8	185.3±33.7	0.89*
LDL-C (mg/dl)	96.2 [83.0 – 131.4]	81.6 [74.8 – 142.2]	0.32**
HDL-C (mg/dl)	56.1±12.1	67.0±14.4	0.033* 0.02^#^
TG (mg/dl)	91.0 [69.0 – 160.0]	79.0 [48.0 – 117.0]	0.13**
Glucose (mg/dl)	86.0±5.6	86.4±6.7	0.87*
Insulin (mIU/ml)	9.4 [7.2 – 13.9]	7.0 [6.1 – 12.9]	0.14**
TG/HDL-C	1.7 [1.2 – 3.8]	1.2 [0.7 – 2.0]	0.049** 0.037^#^
TyG	8.3 [7.8 – 9.4]	8.2 [7.4 – 9.2]	0.16**
TyG-BMI	241.0 [150.3 – 426.4]	215.6 [126.0 – 370.7]	0.43**
HOMA-IR	2.0 [1.5 – 3.1]	1.5 [1.3 – 2.7]	0.15**

Group differences were analyzed using the Student’s t-test (*) for normally distributed variables (presented as mean ± standard deviation) and the Mann–Whitney U test (**) for non-normally distributed variables (presented as median and interquartile range, 25th–75th percentile). p-values (#) were adjusted for BMI and fat mass using analysis of covariance (ANCOVA), with log-transformation of the dependent variable applied to meet model assumptions. AST, aspartate aminotransferase; ALT, alanine aminotransferase; TC, total cholesterol; LDL-C, LDL-cholesterol; HDL-C, HDL-cholesterol; TG, triglycerides; TG/HDL-C, triglycerides to HDL-cholesterol index; TyG, triglycerides glucose index; TyG-BMI, triglycerides glucose-BMI index; HOMA-IR, homeostatic model assessment for insulin resistance.

In adulthood, lipid abnormalities were observed in 12 patients (52.2%) in the SG and 4 patients (36.4%) in the CG (p=0.31). No abnormal insulin or glucose levels were identified in either group.

When analyzing the percentage of patients with elevated calculated indices, high TG/HDL-c values were observed in 8 cases (34.8%) in the SG compared to 1 case (9.1%) in the CG (p=0.12). Elevated TyG index values were found in 17 patients in the SG and 7 in the CG (77.3% vs. 63.6%, p=0.33), while high TyG-BMI values were noted in 18 patients in the SG and 8 in the CG (81.8% vs. 72.7%, p=0.43). In contrast, elevated HOMA-IR was detected in 26% of patients in both groups.

To explore further, we analyzed the longitudinal differences (adolescence to adulthood) for each parameter and compared these changes between the SG and CG. There were no statistically significant differences between the groups in any of these parameters (**Table 5**).

**Table 5:** Comparison of longitudinal changes in biochemical parameters between study and comparison group.

Parameters	Study group	Comparison group	p value
Δ BMI	1.7±3.7	1.3±4.6	0.82*
Δ AST	-4.6±7.9	-3.0±5.4	0.57*
Δ ALT	4.7 [2.8 – 14.3]	3.8 [-0.7 – 7.0]	0.2**
Δ HDL-C	10.3±10.6	12.4±14.0	0.7*
Δ LDL-C	5.6 [-1.2 – 29.6]	1.8 [-3.2 – 14.1]	0.48**
Δ TC	26.7±26.6	31.8±44.5	0.74*
Δ TG	18.3±75.5	9.5±58.0	0.27*
Δ Insulin	0.33 [-5.3 – 3.3]	-0.7 [-4.5 – 1.0]	0.62**
Δ Glucose	2.2±6.4	-6.7±13.0	0.056*
Δ TG/HDL-C	-0.04±2.1	-0.08±1.0	0.96*
Δ TyG	0.1 [-0.04 – 0.5]	-0.2 [-0.5 – 0.6]	0.53**
Δ TyG-BMI	38.5±60.7	5.5±40.3	0.25*
Δ HOMA-IR	0.005 [-1.1 – 0.8]	-0.6 [-1.5 – 0.1]	0.29**

Group differences were analyzed using the Student’s t-test (*) for normally distributed variables (presented as mean ± standard deviation) and the Mann–Whitney U test (**) for non-normally distributed variables (presented as median and interquartile range, 25th–75th percentile). AST, aspartate aminotransferase; ALT, alanine aminotransferase; TC, total cholesterol; LDL-C, LDL-cholesterol; HDL-C, HDL-cholesterol; TG, triglycerides; TG/HDL-C, triglycerides to HDL-cholesterol index; TyG, triglycerides glucose index; TyG-BMI, triglycerides glucose-BMI index; HOMA-IR, homeostatic model assessment for insulin resistance.

**Figure 1:**
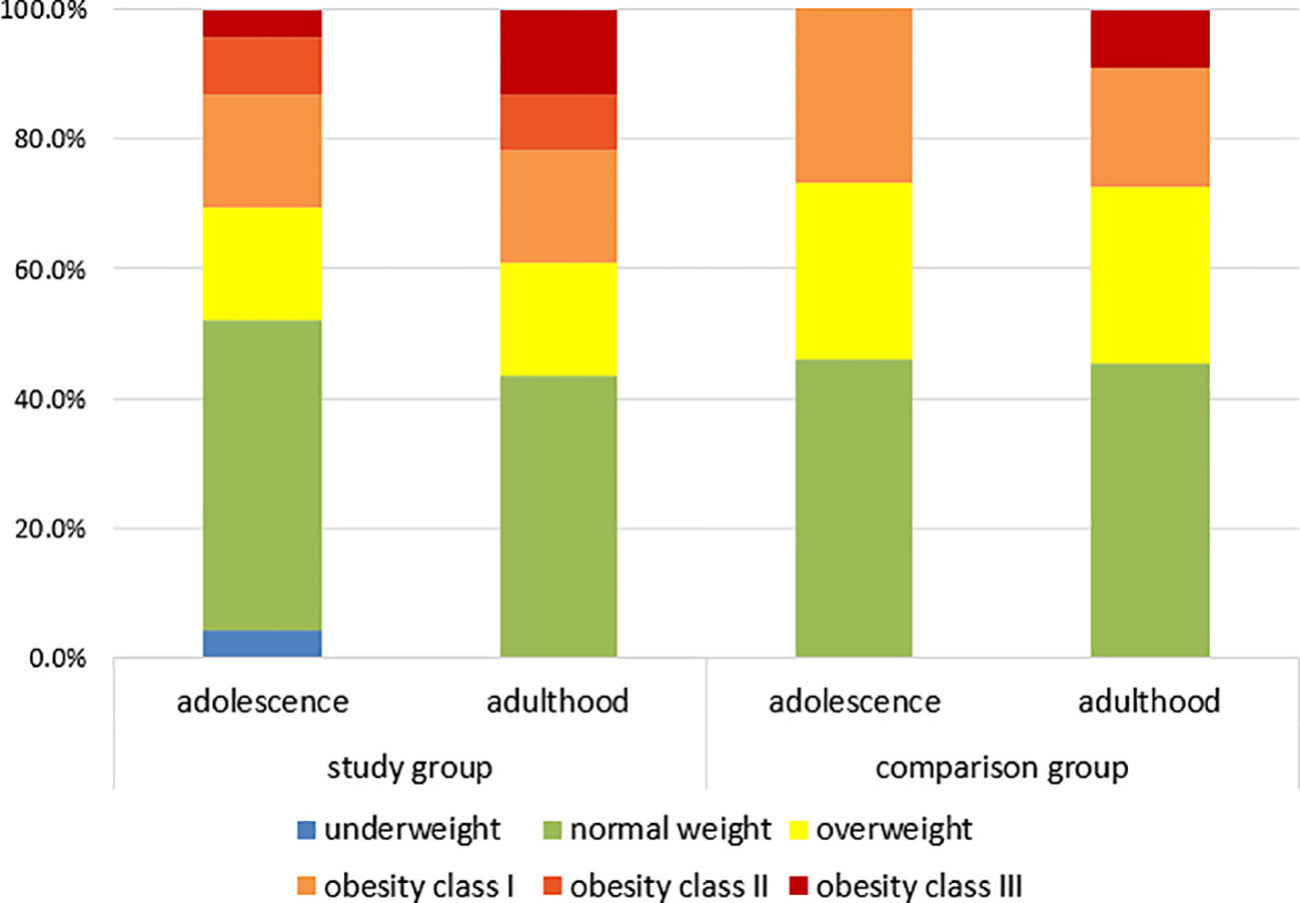
Weight distribution in study group and comparison group. Normal weight: 18.5-24.99 kg/m^2^; overweight: 25-29.9 kg/m^2^, obesity class I: 30-34.9 kg/m^2^; obesity class II: 35–40 kg/m^2^; obesity class III: >40 kg/m^2^.

Although BMI did not differ significantly between groups at the follow-up visit (**Table 4**), the ANCOVA model using change in BMI (ΔBMI) as the dependent variable identified duration of illness as a significant predictor (F(1.29) = 8.64; p = 0.006). As the length of observation may have contributed to the lack of statistical significance in between-group comparisons, we additionally examined weight distribution trends in both groups during adolescence and adulthood (**Figure 1**).

The graphical presentation highlights an increase in overweight and obesity within the SG, rising from 48% during adolescence to 57% in adulthood (p = 0.38). This shift appears to be primarily driven by an increase in the prevalence of class III obesity, which grew from 4.4% in adolescence to 13% in adulthood (p = 0.30). Overall, obesity prevalence in the SG increased from 30.4% to 39.1% (p = 0.38). In contrast, the CG maintained a stable overweight and obesity rate of 54% across both time points.

## 4 Discussion

The objective of this study was to evaluate whether a PCOS diagnosis based on adolescent-specific criteria correlates with an increased risk of metabolic alterations in adulthood. A retrospective data analysis identified 34 women with varying severity of PCOS-related symptoms present in adolescents – 23 meeting the modified adolescent criteria (phenotype A and B of Rotherdam criteria) and 11 not meeting the adolescent criteria of PCOS (phenotype C and D of Rotherdam criteria or at risk of PCOS). Patients were reassessed with blood tests evaluating lipids and carbohydrate metabolism parameters as young adults. General characteristics did not differ significantly between the study and comparison groups during adolescence and adulthood. Key findings include the insulin resistance index (IR) TG/HDL-C, which was higher in the study group, and HDL-C levels, which remained lower. These changes were observed both at the initial evaluation and during reassessment.

In 2017 updated diagnostic criteria for PCOS in adolescent girls were introduced to reduce the overdiagnosis in this population (6). The symptoms outlined in the newly proposed criteria, hyperandrogenism and menstrual disorders, were present respectively in 78% and 88% of our patients, while PCOM was observed in only 38% of cases (**Table 1**). Additionally, elevated testosterone and DHEAS levels in SG in adolescence are consistent with the updated diagnostic criteria. Tay et al. reported a 44% decrease in PCOS diagnoses among adolescents, with the number of cases dropping from 66 under the original criteria to 37 using the updated criteria (11). Similarly, applying these updated criteria in our study resulted in a 33% reduction in PCOS diagnoses among adolescents. However, our findings indicate a slightly smaller reduction in overdiagnosis, likely due to our study’s focus on a strictly selected group from an endocrinology ward rather than a more general population-based sample.

Several studies suggest an increased risk of overweight and obesity in adolescents diagnosed with PCOS, with Bhattacharya and Huang reporting that 40–60% of women with PCOS are overweight or obese (12, 13). Our report revealed similar findings, with 48% of participants in the SG classified as overweight or obese during adolescence, increasing to 57% in adulthood (p=0.38). Tay et al. demonstrated that only the phenotype meeting the updated criteria was significantly associated with a higher long-term BMI increase, while other PCOS phenotypes exhibited BMI trajectories similar to those of participants without PCOS (11). In our study, there was no significant difference in BMI between the groups, nor was there a substantial increase in BMI from adolescence to young adulthood. However, the findings reported by Tay et al. were based on a nearly 20-year longitudinal observation, whereas the maximum follow-up duration in our study was 11 years, with a median of 5.5 years. This aligns with our ANCOVA results, which identified duration of the illness as a significant predictor of BMI changes.

The HOMA-IR is widely used to assess IR in adult populations. DeUgarte et al. reported that HOMA-IR identified IR in 64% of women with PCOS, while Amissi reported a rate of 39.3% (14, 15). In our study, 26% of participants in both the study and comparison groups had abnormal HOMA-IR values in adulthood, with median values of 2.0 in the SG and 1.48 in the CG.

Research by Vasques et al. demonstrates that the TyG index may outperform HOMA-IR in IR assessment, with Yang et al. reporting higher TyG index values in PCOS patients versus controls (16, 17). In our patient cohort, neither the TyG index nor its variant, TyG-BMI, showed significant differences between the study and comparison groups.

Significant findings emerged from the analysis of the TG/HDL-C ratio. The values differed between the two groups and remained higher in the SG during both adolescence and adulthood. The statistically significant differences in log-transformed TG/HDL-C between the study and comparison groups persisted after adjusting for BMI in adolescents, and for both BMI and fat mass in adults, suggesting a group-related effect. In adults, BMI independently contributed to TG/HDL-C levels, highlighting the greater influence of body weight in this age group, whereas in adolescents, the group effect appeared to be independent of BMI. Our findings, in conjunction with the available literature, highlight the potential of using TG/HDL-C as a marker for monitoring IR risk in patients with PCOS from adolescence, especially given that a significant correlation between this ratio and insulin levels was observed at this age (p=0.005, R=0.61).

Emerging evidence suggests that an elevated TG/HDL-C ratio may serve as an early biomarker for cardiometabolic complications in women with PCOS, a population with an increased risk of such conditions (18). Wan et al. further reported a significantly higher incidence of cardiovascular disease (CVD) in women with PCOS across all age groups (10–54 years), with the number of PCOS-associated CVD cases rising from 102,530 in 1990 to 235,560 in 2019 (19). However, we recognize the need to establish a standardized consensus on cut-off points, as these may vary depending on the specific condition or clinical context.

Findings on body composition in patients with PCOS remain inconsistent. While Sun et al. reported increased abdominal fat accumulation, Zakeri et al. found no significant differences. Similarly, our study did not demonstrate any statistically significant differences in body composition between the study and control groups. Moreover, fat mass did not show predictive value for key metabolic outcomes that reached statistical significance in our analyses, including HDL-C and TG/HDL-C levels.

Insulin resistance is frequently accompanied by dyslipidemia, including elevated TG and LDL-C levels and decreased HDL-C levels. Although dyslipidemia is not a diagnostic criterion for PCOS, it is a recognized metabolic phenotype, with an estimated prevalence of 70% in PCOS populations (20). Zhao et al. found that all four PCOS phenotypes exhibit increased levels of very low-density lipoprotein (VLDL), LDL-C, fatty acids, and unsaturated fatty acids, with higher LDL-C and lower HDL-C in PCOS patients compared to controls (21). In our cohort, lipid abnormalities in adulthood were observed in 43.5% of patients in the SG and 36.4% in the CG (p=0.31). In comparison with the study by Zhao, we demonstrated differing HDL-C values, which were lower in the study group. These results were observed both in the adolescent cohort and in adulthood.

Previous studies indicate that in non-obese women with PCOS, low HDL-C levels and elevated TG/HDL-C ratios are not solely a consequence of obesity or insulin resistance but may represent intrinsic features of the syndrome itself (21). This dysregulation of lipid metabolism appears to be driven by the underlying pathophysiology of PCOS, potentially involving hormonal imbalances, chronic low-grade inflammation, or other inherent metabolic disturbances (22, 23). These findings highlight an important area for further investigation, particularly in studies with larger cohorts to better understand the metabolic profile of PCOS independent of obesity.

## 5 Limitations of the study

The present study has several limitations that should be considered when interpreting the findings. The cohort size was relatively small (n=34), which may restrict the generalizability of the results to a broader adolescent PCOS population. *Post hoc* power analysis based on group-level data indicated that a power greater than 0.80 was achieved for several comparisons involving adolescent parameters, including TG/HDL-C, HDL-C, triglycerides, and glucose. While these findings suggest sufficient sensitivity to detect meaningful effects in these cases, the results also provide guidance on the minimum sample sizes needed to ensure adequate power in future studies. Additionally, the follow-up period (median: 5.5 years) may not be sufficient to fully capture the long-term metabolic consequences associated with the syndrome. Furthermore, as participants were recruited from an endocrinology department, the findings may not be fully representative of all adolescents with PCOS, particularly those with milder phenotypes. While this allows for in-depth characterization of such cases, it may limit the external validity of the findings, and caution is warranted when generalizing the results to broader, more heterogeneous populations. Diet, physical activity, and socioeconomic status were not included in the analysis, which may influence metabolic outcomes. The study did not apply Bonferroni correction or other familywise error rate adjustments for multiple comparisons. Given the exploratory nature of the analysis and the small cohort size, applying such corrections could have substantially increased the risk of Type II errors, potentially obscuring meaningful trends. However, the lack of correction requires that the results be interpreted with caution. These limitations highlight the need for further large-scale, long-term studies to validate and expand upon these findings.

## 6 Conclusion

This study provides preliminary observations regarding the application of updated diagnostic criteria for PCOS in adolescent girls and their potential role in identifying individuals at risk for long-term metabolic abnormalities. The analysis of insulin resistance markers, particularly the TG/HDL-C ratio, offers insights into possible early metabolic changes in this population. Observed abnormalities in HDL-C levels and TG/HDL-C ratios during adolescence appeared to persist into adulthood without a significant increase in body weight.

These findings indicate that lipid abnormalities might be a feature of PCOS independent of traditional metabolic factors such as obesity or insulin resistance. However, further research involving larger cohorts is necessary to confirm these associations and to better understand the clinical significance of lipid profile alterations in adolescent PCOS populations.

## Data Availability

The raw data supporting the conclusions of this article will be made available by the authors, without undue reservation.

## References

[1] Guo F Gong Z Fernando T Zhang L Zhu X Shi Y. The lipid profiles in different characteristics of women with PCOS and the interaction between dyslipidemia and metabolic disorder states: a retrospective study in Chinese population. Front Endocrinol (Lausanne). (2022) 13:892125. doi: 10.3389/fendo.2022.892125 35860700 PMC9289193

[2] Chang S Dunaif A. Diagnosis of polycystic ovary syndrome: which criteria to use and when? Endocrinol Metab Clin North Am. (2021) 50:11–23. doi: 10.1016/j.ecl.2020.10.002 33518179 PMC7860982

[3] Altintas KZ Dilbaz B Cirik DA Ozelci R Zengin T Erginay ON. The incidence of metabolic syndrome in adolescents with different phenotypes of PCOS. Ginekol Pol. (2017) 88:289–95. doi: 10.5603/GP.a2017.0055 28727126

[4] Foryś E Baran A Dziurdzia A Jarosz-Wójcik E Matusik P Gawlik A. Czy zaburzenia miesiączkowania u dorastających dziewcząt są związane z zaburzeniami metabolicznymi? Pediatr Endocrinol Diabetes Metab. (2023) 29:75–82. doi: 10.5114/pedm.2023.125364 37728458 PMC10411084

[5] Witchel SF Oberfield SE Peña AS. Polycystic ovary syndrome: pathophysiology, presentation, and treatment with emphasis on adolescent girls. J Endocr Soc. (2019) 3:1545–73. doi: 10.1210/js.2019-00078 PMC6676075 31384717

[6] Ibáñez L Oberfield SE Witchel S Auchus RJ Chang RJ Codner E. An international consortium update: pathophysiology, diagnosis, and treatment of polycystic ovarian syndrome in adolescence. Horm Res Paediatr. (2017) 88:371–95. doi: 10.1159/000479371 29156452

[7] Teede HJ Tay CT Laven J Dokras A Moran LJ Piltonen TT. Recommendations from the 2023 international evidence-based guideline for the assessment and management of polycystic ovary syndrome. Fertil Steril. (2023) 120:767–93. doi: 10.1016/j.fertnstert.2023.07.025 37589624

[8] Anagnostis P Tarlatzis BC Kauffman RP. Polycystic ovarian syndrome (PCOS): long-term metabolic consequences. Metabolism. (2018) 86:33–43. doi: 10.1016/j.metabol.2017.09.016 29024702

[9] McLaughlin T Abbasi F Cheal K Chu J Lamendola C Reaven G. Use of metabolic markers to identify overweight individuals who are insulin resistant. Ann Intern Med. (2003) 139:802–9. doi: 10.7326/0003-4819-139-10-200311180-00007 14623617

[10] Vasques AC Novaes FS de Oliveira Mda S Souza JAM Yamanaka A Pareja JC. TyG index performs better than HOMA in a Brazilian population: a hyperglycemic clamp validated study. Diabetes Res Clin Pract. (2011) 93:e98–e100. doi: 10.1016/j.diabres.2011.05.030 21665314

[11] Tay CT Hart RJ Hickey M Moran LJ Earnest A Doherty DA. Updated adolescent diagnostic criteria for polycystic ovary syndrome: impact on prevalence and longitudinal body mass index trajectories from birth to adulthood. BMC Med. (2020) 18:389. doi: 10.1186/s12916-020-01861-x 33302955 PMC7731536

[12] Huang J Ni R Chen X Huang L Mo Y Yang D. Metabolic abnormalities in adolescents with polycystic ovary syndrome in south China. Reprod Biol Endocrinol. (2010) 8:142. doi: 10.1186/1477-7827-8-14 21083920 PMC2994875

[13] Akshaya S Bhattacharya R. Comparative study of clinical profile of lean and obese polycystic ovary syndrome women. Int J Reprod Contracept Obstet Gynecol. (2017) 5:2530–3. doi: 10.18203/2320-1770.ijrcog20162173

[14] DeUgarte CM Bartolucci AA Azziz R. Prevalence of insulin resistance in the polycystic ovary syndrome using the homeostasis model assessment. Fertil Steril. (2005) 83:1454–60. doi: 10.1016/j.fertnstert.2004.11.070 15866584

[15] Amisi CA. Markers of insulin resistance in polycystic ovary syndrome women: an update. World J Diabetes. (2022) 13:129–49. doi: 10.4239/wjd.v13.i3.129 PMC8984569 35432749

[16] Yang H Chen Y Liu C. Triglyceride-glucose index is associated with metabolic syndrome in women with polycystic ovary syndrome. Gynecol Endocrinol. (2023) 39:2172154. doi: 10.1080/09513590.2023.2172154 36708155

[17] Reckziegel MB Nepomuceno P MaChado T Renner JDP Pohl HH Nogueira-de-Almeida CA. The triglyceride-glucose index as an indicator of insulin resistance and cardiometabolic risk in Brazilian adolescents. Arch Endocrinol Metab. (2023) 67:153–61. doi: 10.20945/2359-3997000000506 PMC10689041 36651702

[18] Jabczyk M Nowak J Jagielski P Hudzik B Kulik-Kupka K Włodarczyk A. Metabolic deregulations in patients with polycystic ovary syndrome. Metabolites. (2023) 13:202. doi: 10.3390/metabo13020302 36837921 PMC9964823

[19] Wan Z Zhao J Ye Y Sun Z Li K Chen Y. Risk and incidence of cardiovascular disease associated with polycystic ovary syndrome. Eur J Prev Cardiol. (2024) 31:1560–70. doi: 10.1093/eurjpc/zwae066 38373259

[20] Chen W Pang Y. Metabolic syndrome and PCOS: pathogenesis and the role of metabolites. Metabolites. (2021) 11. doi: 10.3390/metabo11120869 PMC8709086 34940628

[21] Zhao Y Fu L Li R. Metabolic profiles characterizing different phenotypes of polycystic ovary syndrome: plasma metabolomics analysis. BMC Med. (2012) 10:153. doi: 10.1186/1741-7015-10-153 23198915 PMC3599233

[22] Butler AE Moin ASM Reiner Ž Sathyapalan T Jamialahmadi T Sahebkar A. High density lipoprotein-associated proteins in non-obese women with and without polycystic ovary syndrome. Front Endocrinol (Lausanne). (2023) 14:1117761. doi: 10.3389/fendo.2023.1117761 37181037 PMC10171110

[23] Diamanti-Kandarakis E Papavassiliou AG Kandarakis SA Chrousos GP. Pathophysiology and types of dyslipidemia in PCOS. Trends Endocrinol Metab. (2007) 18:280–5. doi: 10.1016/j.tem.2007.07.004 17692530

